# New Method for the Determination of Lamotrigine in Human Saliva Using SPE-LC-DAD

**DOI:** 10.3390/molecules30153237

**Published:** 2025-08-01

**Authors:** Ewelina Dziurkowska, Aleksandra Michalak, Alina Plenis, Maciej Dziurkowski

**Affiliations:** 1 Department of Analytical Chemistry, Medical University of Gdansk, Gen. J. Hallera 107, 80-416 Gdansk, Poland; olamichalak14@gmail.com (A.M.); alina.plenis@gumed.edu.pl (A.P.); 2 Hospital for Nervous and Mental Diseases, Skarszewska 7, 83-200 Starogard Gdanski, Poland; mdart2002@yahoo.com

**Keywords:** saliva, lamotrigine, solid-phase extraction, liquid chromatography, bipolar disorder

## Abstract

(1) Background: The concentration of lamotrigine, an antiepileptic drug very often used in bipolar disorder, is most often determined in the blood, with many inconveniences. An alternative may be to use saliva as a diagnostic material for this purpose. The development of a method to determine lamotrigine in saliva as a biological material significantly improves patient comfort during sampling. The developed method uses solid-phase extraction for the isolation of the drug from saliva for the first time. (2) Methods: This study aimed to develop a method to determine lamotrigine in saliva using solid-phase extraction (SPE) for isolation and liquid chromatography with a diode array detector (LC-DAD) for quantitative analysis. (3) Results: The method was validated by determining its linearity in the concentration range 10–2000 ng/mL (R^2^ > 0.99), and the intra- and inter-day precision expressed as coefficient of variation (CV%) did not exceed 15%. (4) Conclusions: The developed method was used to determine the salivary concentration of lamotrigine in patients treated with the studied compound, confirming its usefulness in bipolar disorder (BD).

## 1. Introduction

Bipolar disorder (BD) is characterised by cyclical recurrent fluctuations in mood, energy, and behaviour that accompany the patient with varying degrees of severity throughout life. Bipolar affective disorder can be triggered by both genetic and environmental factors. It is accompanied by dysregulation of neurotransmitters, neurohormones, and secondary messenger systems in the brain. Due to the complexity of the symptoms, as well as the high rate of suicidal thoughts and behaviour, BD is a difficult disease to diagnose and subsequently treat. BD therapy is primarily aimed at improving the patient’s functioning, especially by reducing the number and severity of depressive episodes, as well as manic and hypomanic episodes. Therefore, depending on the patient’s condition, the main medications used for this condition are lithium, antipsychotics, and antiepileptic drugs, when their mood-stabilising effects are exploited [1].

Due to the multiplicity of symptoms, therapy and dosage in BD must be individualised. It can be an additional challenge if a patient has rapid cycling bipolar disorder. This term was introduced to treatment in 1974 and describes patients who respond poorly to treatment and, in addition, have a frequency of a minimum of four episodes per year. This type of BD affects women more often than men and is more often accompanied by suicidal thoughts and attempts. A rapid change from depressive mood to mania is also frequently observed, particularly when antidepressants are used. Therefore, the standard treatment in this case is the administration of mood stabilisers [2].

Lamotrigine, which is an antiepileptic drug, is used in bipolar disorder because of its mood-stabilising effects. Some studies show that lamotrigine, along with quetiapine and antidepressants, is the most commonly used drug in BD, particularly in women and people over 40 years of age [3]. The primary mechanism of action of lamotrigine is the inhibition of voltage-gated sodium channels, thereby blocking continuous repetitive excitation of neurons and prolonging the repolarisation time of cell membranes. Lamotrigine has inhibitory properties on glutamate release [1]. In addition, an in vivo study has shown that it inhibits R-type (residual) calcium currents, which may further contribute to the anticonvulsant property of this active substance [4].

Lamotrigine is rapidly and completely absorbed and its bioavailability is estimated to be 98%. Maximum concentration after a dose of the drug is reached between 1.4 and 4.8 h. Lamotrigine is approximately 55% bound by plasma proteins. The metabolism of lamotrigine occurs in the liver; conjugation with glucuronic acid produces inactive metabolites. Hepatic methyltransferase in turn produces 2-methyl-lamotrigine. Lamotrigine is excreted in both urine and faeces [1].

Lamotrigine is a relatively safe drug and can therefore be used in children over 2 years of age. However, side effects can also occur here. Some of these include skin rash and headache. Usually, the rash is of mild intensity and resolves spontaneously. However, severe, life-threatening forms may occur, such as Stevens–Johnson syndrome (acute necrosis of the epidermis and mucous membranes covering less than 10% of the body surface) or hypersensitivity syndrome (HSS). The risk of skin reaction is also correlated with age. It is more likely to occur in children than in adults. Another major risk associated with lamotrigine treatment is suicidal thoughts and behaviour. Close observation of patients taking this drug is necessary. An important interaction is the interaction between lamotrigine and oral hormonal contraception. Their concomitant use without appropriate consultation may be associated with an increase in the side effects of lamotrigine, as well as a decrease in the effectiveness of hormonal contraception [1].

When using lamotrigine in BD, it is necessary to control its concentration in the body. This is mainly due to the fact that during an exacerbation of the symptoms of the disease, it is very often necessary to use several preparations simultaneously, at least periodically. In such a situation, side effects may be exacerbated. And monitoring of drug concentrations will help exclude possible intoxication. Little information in the literature describes therapeutic concentrations in BD. It indicates that the most commonly determined lamotrigine concentrations are lower than those determined during treatment of epilepsy [5]. Therefore, it is extremely important to determine lamotrigine levels in the body.

There is a correlation between drug concentration in blood and therapeutic effects, but there is no satisfactory correlation between dose and drug concentration in blood. Most commonly, lamotrigine is determined in blood [3,6,7,8,9,10,11,12,13]. However, alternative biological matrices may include urine [11,12], saliva [7,8,14,15], breast milk [16], and hair [7,14]. Monitoring of lamotrigine therapy is specifically recommended if the patient is pregnant, breastfeeding, or taking other medications (primarily antiepileptic drugs and hormonal contraception). Control of lamotrigine concentrations is also advisable in children, as its clearance is higher in this group of patients compared with adults [6,7,16]. There is a high correlation between salivary lamotrigine concentrations and serum concentrations of this drug. Data suggest that saliva monitoring may play a role in controlling lamotrigine concentrations in both adult and pediatric patients [8,15,17,18,19].

As mentioned above, lamotrigine in BD is mostly used in polytherapy, so control of the drug concentration is advisable to reduce the severity of side effects and to avoid toxic effects when drugs affecting metabolism are used concomitantly. Determination of drug levels in saliva allows rapid and non-invasive sampling, performed by the patient themself. In addition, it allows frequent monitoring of drug levels whilst avoiding uncomfortable blood sampling, which requires pricking.

Some of the most commonly used methods for the isolation of lamotrigine from biological matrices are liquid–liquid extraction (LLE) [7,8,15,20,21] and solid-phase extraction (SPE) [9,10,11,13]. Protein precipitation [14,22,23] and solidified floating organic drop microextraction [12], which is a modified form of LLE and microextraction by packed sorbent [17], have also been used. Quantitative analysis was performed by liquid chromatography coupled with MS detection [7,9,10,14,21,23] or UV (DAD) [8,12,13,15,17,18,20].

Due to the need to select the dose individually for the patient, as well as to control the severity of adverse effects and possible dosage changes, there is a need to develop a method to rapidly monitor lamotrigine levels in the body. The readily available biological material of saliva provides a convenient way to collect a sample for analysis. However, there are no standardised procedures for the determination of lamotrigine concentrations in this biological material. In addition, literature data indicate that studies of lamotrigine concentrations in saliva have so far mostly been conducted on people being treated for epilepsy. As mentioned above, the therapeutic doses used in BD are usually lower, but there are few data on the determination of lamotrigine concentrations in blood in this group of patients. In addition, lamotrigine has so far been isolated from saliva exclusively using LLE, an extraction that requires toxic solvents. SPE has not been used to date. Therefore this study aimed to develop a rapid and reliable method to determine lamotrigine concentration in a small volume of human saliva using solid-phase extraction and liquid chromatography with DAD detection.

## 2. Results

### 2.1. Chromatographic Analysis

**Figure 1:**
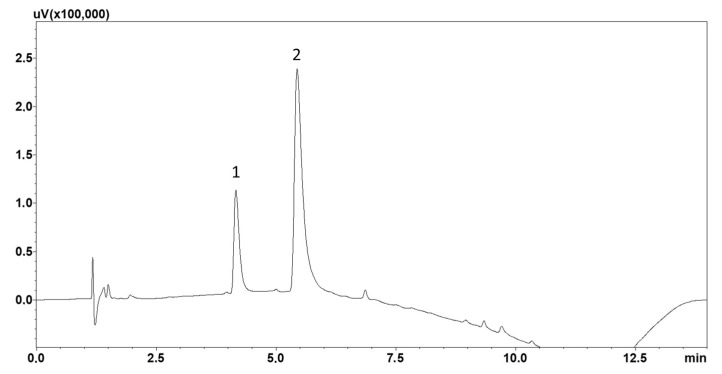
Chromatogram of the standard solutions obtained by optimised LC: (1) lamotrigine; (2) chlordiazepoxide (IS).

When optimising the separation process, an appropriate mobile phase composition and flow rate were chosen. The basic mobile phase composition was acetonitrile and a 0.1% formic acid solution. The addition of 0.1% triethylamine was also investigated; however, the addition of the latter negatively affected the peak shape. Based on retention time analysis of the standard solutions and chromatographic analysis of blank saliva samples, it was found that the best separation of lamotrigine and IS from other matrix elements was obtained using gradient elution, details of which are presented in Section 4.1. Detection of the compounds was carried out at 248 nm. An example chromatogram showing the analysis of standard solutions performed under optimised separation conditions is shown in Figure 1.

### 2.2. Solid-Phase Extraction

**Figure 2:**
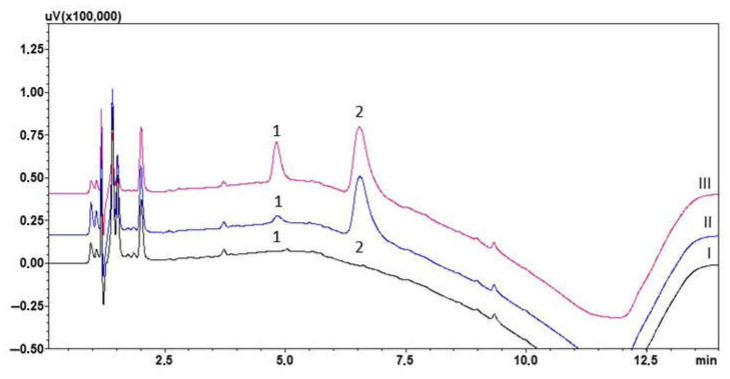
Chromatogram of saliva sample extract obtained spiked with 1000 ng/mL (III) or 200 ng/mL (II) of lamotrigine and blank saliva sample extract (I). (1) Lamotrigine; (2) chlordiazepoxide (IS).

During the optimisation process, the solutions used to dilute the saliva sample, the appropriate SPE cartridge, and the solution mixtures used during the extraction procedure were selected. Saliva was diluted with 0.1 M sodium acetate solution or 0.1 M potassium hydrogen phosphate solution. In both cases, the volume ratio of saliva to dilution solution was 1:1. A suitable SPE sorbent was then selected to ensure both optimum purification of the sample from the ballast from the biological matrix and maximum efficiency of the extraction process. The SPE cartridges analysed included StrataX (Phenomenex) and ABN (Biotage). Independent of the columns used, a 10% methanol solution was used to wash the cartridge. On the other hand, elution of the analyte and IS was performed with methanol. Analysis of the chromatograms of the blank saliva samples extracted under the conditions described showed that the ABN sorbent did not allow complete removal of the ballast and a peak originating from the matrix was observed during IS retention. The final decision was to dilute the saliva samples with 0.1 M K_2_HPO_4_ solution and Strata-X columns. The developed procedure was then applied to the analysis of loaded saliva samples. Figure 2 shows the chromatogram of blank saliva samples and extracts of 200 and 1000 ng/mL lamotrigine-spiked saliva samples.

### 2.3. Method Validation

The next stage of the study was to validate the developed method. Its linearity was determined in the range 10–2000 ng/mL. The R^2^ coefficient exceeded 0.99 (R^2^ = 0.9996), confirming that the developed method meets the requirements of the EMA (European Medicines Agency) in the tested concentration range. The limit of quantification was set at 10 ng/mL. Detailed measurement results are presented in Table 1. They indicate that the method is precise, as evidenced by the CV value, which did not exceed 15% for all concentrations tested. The highest CV value was 14.39% for the precision between days for the concentration of 10 ng/mL, which is also the LLOQ (Lower Limit of Quantification) concentration. According to EMA guidelines, this value should not exceed 20%.

#### 2.3.1. Extraction and Absolute Recovery

Four concentrations of lamotrigine QC (Quality Control) were investigated to determine extraction recovery and absolute recovery. The detailed results are shown in Table 2. They indicate that extraction recovery, which was determined as a comparison of the peak area fields of lamotrigine extracts to the area fields of blank saliva samples spiked after the extraction process, meets the EMA guidelines (>50%), as it falls between 93.63 and 101.82%. Similarly, absolute recovery, determined as a comparison of the areas of peaks obtained after the extraction process to the areas of six neat standards at each concentration, which ranged between 90.95 and 100.03%, met the EMA guidelines for all concentrations tested.

#### 2.3.2. Stability

The lamotrigine stability study included a freeze–thaw test (−21 °C) and an autosampler storage test of lamotrigine extracts at 8 °C. The results of the study are summarised in Table 2 and presented as % loss of concentration determined on the first day of the study. The results obtained indicate that lemotrigine was stable under the storage conditions tested for all concentrations.

### 2.4. Clinical Application

**Figure 3:**
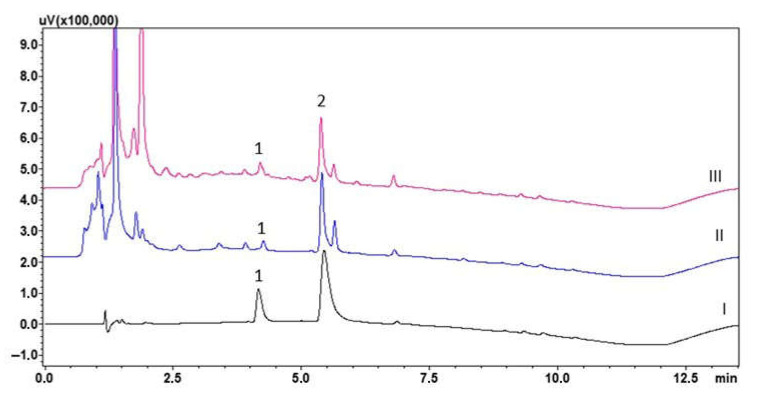
Chromatogram of saliva extracts of patients 5 (II) and 7 (III) treated with lamotrigine (50 mg/day) and blank saliva sample extract (I): (1) lamotrigine; (2) chlordiazepoxide (IS).

The method developed was applied to the determination of lamotrigine in seven saliva samples from patients treated with the aforementioned drug at a dose of 50 mg once daily, overnight. Example chromatograms of the patients’ saliva extracts are shown in Figure 3, and the results obtained are described in Table 3.

## 3. Discussion

Determination of lamotrigine levels in BD is not common, although due to the combination therapy used in this condition, control of drug concentrations could help to reduce side effects. Previous literature data indicate that lamotrigine concentrations in the body are mainly determined in blood [3,6,7,8,9,10,11,12,13,14], with only a few data points reporting studies in other biological materials. Although there is a correlation between lamotrigine concentrations in blood and saliva, this compound has rarely been determined in this readily available diagnostic material. In addition, the extraction that has been used to date for the isolation of lamotrigine from saliva has been LLE [7,8,14,15]. Although it allows good purification of the sample from matrix residues, it always requires volatile and toxic solvents, and an emulsion often forms at the interface, which can affect the precision of the assay.

As we know, the study presented here is the first in which SPE has been used to isolate lamotrigine from this biological material. In addition, the sample volume is small (200 µL), which is expected to allow it to be collected also from people suffering from dry mouth. The method developed uses Strata X columns. For blood, lamotrigine isolation was performed using HLB (Hydrophilic-Lipophilic Balanced) columns. Both types of sorbents are designed for lipophilic compounds. The use of SPE for the isolation of lamotrigine allowed us to achieve a high analyte recovery that exceeded 93%, which overlaps with [7] or slightly exceeds [8] the results obtained with LLE. However, as already mentioned, SPE avoids the use of volatile and toxic solvents, making it perfectly in line with the green chemistry trend.

The developed method was characterised by linearity in the concentration range 10–2000 ng/mL. Methods reported in the literature that isolated lamotrigine from saliva were most often characterised by linearity in the concentration range between 0.5 and 20 µg/mL [7,14]. It is possible that the concentration range studied was because the study involved epileptic patients with a higher dose of the drug than BD. From the literature data available to us, lamotrigine has not been determined in the saliva of patients with BD to date, although lamotrigine is one of the more commonly prescribed drugs in BD, particularly among women [2,5]. Therefore, we aimed to lower the limit of quantification. Our assumption is also based on the fact that BD sufferers often discontinue their medication or modify the dosage without following the advice of the treating physician. As a result, the expected improvement in the patient’s condition is not observed.

The method developed has a high precision of determination as evidenced by low CV values below 10.2%. Only in the case of LLOQ, for which the EMA allows a CV of 20%, the inter- and intra-day precision was below 14.4%. However, it should be emphasised that in the case of lamotrigine determination in saliva, no method had such a low LLOQ.

Based on literature data confirming the correlation of lamotrigine concentrations in the saliva and blood of people with epilepsy [8,15,17,18,19], we focused on the development and validation of a method that allows the determination of lamotrigine levels in the saliva of people treated with the mentioned drug using SPE to isolate the compound. Due to the lower doses used in BD, we were interested in which lamotrigine concentrations would be observed in the saliva of BD subjects many hours after drug administration. For our patients, samples were collected approximately 14 h after drug administration. Patients received the drug in the evening around 8 p.m., while samples were collected in the morning around 10 a.m. We were able to determine lamotrigine concentrations in all samples tested. The range of lamotrigine concentrations determined in saliva was between 228.6 and 1058.00 ng/mL. All patients received the same dose at the same time (50 mg in the evening). Except for one patient, the drug concentration did not exceed 630 ng/mL. Only in one case did the drug level exceed 1000 ng/mL, which may be indicative of slower metabolism. In our study, we also did not take into account the effect of the patient’s weight and other drugs used, as well as the duration of therapy.

Literature data indicate that the mean blood concentration of lamotrigine determined in BD patients treated with 200 mg of the drug per day ranges between 5.30 ± 3.61 and 8.07 ± 3.71 mg/mL, depending on the time elapsed since the start of therapy [22]. Lower values were observed with longer administration of the drug. Given that the patients in whom we determined lamotrigine levels in saliva used a lower dose of the drug, the concentrations we determined were proportionally lower than those determined in blood. According to our data, this study is the first to determine lamotrigine concentrations in BD patients where the drug was used as a normothymic, and hence the dose used was lower than for epilepsy. The few data in the literature indicate that when such a low dose of lamotrigine is used, the determined salivary concentration coincides with our observations [7].

## 4. Materials and Methods

### 4.1. Chemicals and Solvents

Stock solution (1 mg/mL) of lamotrigine was obtained from Sigma-Aldrich (St. Louis, MO, USA). The internal standard (IS) chlordiazepoxide (Polfa Tarchomin, Warsaw, Poland) was prepared by dissolving 10 mg of the substance in 1 mL of methanol. Methanol and acetonitrile (both of HPLC gradient grade) in addition to formic acid (98–100%) and potassium hydrogen phosphate (K_2_HPO_4_) (both reagent-grade) were obtained from POCh (Gliwice, Poland). Water was purified by an Ultra-Toc/UV system, Hydrolab (Straszyn, Poland). For solid-phase extraction, Strata–X (30 mg/3 mL) columns from Phenomenex (Torrance, CA, USA) were used.

### 4.2. Chromatographic Analysis

The separation was carried out using a Nexera XR UHPLC liquid chromatograph (Shimadzu, Kyoto, Japan) consisting of a CBM-20Alite control system, LC-30 AD pump, CTO-20AC thermostat, SIL-30AC autosampler, SPD-M30A UV-VIS detector with diode array, and SPD-M30A high-sensitivity measuring cell (85 mm). The stationary phase was a Nucleosil 100–5 chromatography column (C18; 125 mm × 4 mm, 5 µm; Knauer, Berlin, Germany) with a pre-column. The column temperature was 35 °C. A mixture of acetonitrile and 0.1% formic acid was used as the mobile phase at a flow rate of 1 mL/min, with a linear gradient starting with 25% acetonitrile and ending with 90% acetonitrile in 9 min. The total analysis time was 14 min.

### 4.3. Standard and Quality Control (QC) Sample Preparation

The stock solutions were used for the preparation of calibrators and QC samples. Working solutions of analytes were prepared by diluting stock solutions of lamotrigine (1 mg/mL) and IS (10 mg/mL) in methanol. All solutions were stored at −21 °C. Calibration samples were prepared by spiking the blank saliva samples with 20 µL of IS (10 µg/mL) and an appropriate amount of lamotrigine working solutions so that the final concentration of lamotrigine was 10, 50, 200, 500, 1000, and 2000 ng/mL. QC samples were prepared as follows: blank saliva samples were spiked with 20 µL of IS (10 µg/mL) and an appropriate amount of lamotrigine working solution so that the final lamotrigine concentration was 10, 100, 800, and 1500 ng/mL.

### 4.4. Saliva Sample Collection

Before the process, volunteers were required to refrain from eating for at least half an hour and to rinse their mouths with water 10 min before sampling. To collect a saliva sample, participants placed Salivettes^®^ (Sarstedt, Nümbrecht, Germany) in their mouths for 2 min, which were then centrifuged at 8000 rpm for 5 min. The resulting saliva was stored at −21 °C until analysis.

### 4.5. Extraction Procedure

Frozen saliva samples were thawed and centrifuged. Then 200 µL blank saliva samples were collected and transferred to plastic tubes. An appropriate amount of lamotrigine and IS was added and diluted with 200 μL of 0.1 M K_2_HPO_4_. The whole mixture was mixed and applied to SPE columns, which had been pre-conditioned with 0.5 mL of methanol and 0.5 mL of water. After the samples were applied, the cartridge was washed with 500 µL of 10% methanol and dried under reduced pressure for 10 min. For elution of lamotrigine and IS, 500 μL of methanol was used and evaporated at 37 °C. The dry residue was reconstituted in 100 µL of a mixture of acetonitrile and 0.1% formic acid (1:9; *v*:*v*). The samples were then vortex-mixed and transferred to autosampler vials, from which 10 µL was taken, injected onto a chromatography column, and analysed under the conditions described above.

### 4.6. Validation

The validation process was performed in accordance with the EMA guidelines [24].

#### 4.6.1. Linearity

The linearity of the method was determined by analysing four calibration curves in the concentration range 10–2000 ng/mL, analysed on four different days. Coefficient of determination (R^2^) > 0.99 and residuals of 15% at each concentration were considered acceptable result. Only at the lower limit of quantification (LLOQ) were 20% residuals accepted.

#### 4.6.2. Intra- and Inter-Day Precision

The precision of the method was expressed in terms of imprecision by calculating the CV% of pooled within-day and between-day values. Imprecision was deemed acceptable for CV 15%. The precision of the method was measured for four concentrations of lamotrigine (10, 100, 800, and 1500 ng/mL). For intra-day precision, each concentration was analysed in five replicates within one day. The inter-day precision was determined by performing five analyses of each concentration for four consecutive days (*n* = 20).

#### 4.6.3. Lower Limit of Quantification (LLOQ)

LLOQ was defined as the lowest concentration that could be quantified with a signal-to-noise ratio of 10, adequate precision (coefficient of variation (CV%) <20%), and accuracy (target concentration 20%). LLOQ was evaluated by analysing five replicates.

#### 4.6.4. Selectivity

The selectivity of the method was determined by analysing blank saliva samples from 10 healthy volunteers. After collection, saliva samples were subjected to extraction as described in Section 4.5 and chromatographic analysis as described in Section 4.2. The presence of endogenous substances at retention times corresponding to the analysed compounds was then excluded. The absence of interferents allowed us to conclude that the method was selective.

#### 4.6.5. Extraction and Absolute Recovery

The extraction and absolute recovery were determined for four concentrations of lamotrigine (10, 100, 800, and 1500 ng/mL). Each concentration was tested six times. The extraction recovery was established by comparing the areas of the extracted analyte with those obtained from ten blank post-spiked samples. The absolute recovery was performed by comparing the peak areas of the extracted analyte with the peak areas of the six neat standards. In both cases, an acceptable result was considered to be a value exceeding 50%.

#### 4.6.6. Stability

The stability of lamotrigine was tested for four concentrations (10, 100, 800, and 1500 ng/mL) by carrying out a freeze–thaw test, with saliva samples stored at −21 °C, and while samples were kept at 8 °C. Each concentration was analysed three times. If the concentration of lamotrigine decreased by less than 15% under the given storage conditions, it was considered to be stable.

### 4.7. Clinical Application

The developed method was applied to determine the concentration of lamotrigine in saliva obtained from patients treated with 50 mg once a day, overnight. Saliva samples were obtained from 7 patients in the morning about 10 a.m. The samples were collected from patients of the Nervous and Mentally Ill Hospital in Starogard Gdanski (Poland). The study protocol was approved by the ethical committee of the Medical University of Gdansk, Poland (NKBBN/393/2021; 25 June 2021).

## 5. Conclusions

A rapid and efficient method for the determination of lamotrigine concentrations in human saliva was developed. Solid-phase extraction was used for the isolation of the compound with an efficiency of more than 93%. The extraction procedure involved diluting 200 µL of the saliva sample with K_2_HPO_4_ and applying it onto SPE columns with Strata-X sorbent. The bed was washed with 10% methanol, followed by drying, and lamotrigine and IS were eluted with methanol. Quantitative analysis was performed by LC-DAD, using a C18-expressing column as the stationary phase. On the other hand, the mobile phase was a mixture of acetonitrile and 0.1% formic acid solution. The developed method was linear in the concentration range between 10 and 2000 ng/mL. It was characterised by high intra- and inter-day precision. Lamotrigine was stable under the conditions tested. The developed SPE-LC-DAD method was also used to determine the concentration of the drug in the saliva of the patients. Lamotrigine concentrations were determined in all samples tested. Therefore, it can be assumed that the developed method can be used to monitor the concentration of lamotrigine in the saliva of patients.

## Figures and Tables

**Table 1:** Calibration curve parameters for the developed method.

Calibration Curve *y* = *ax* + *b* (*n* = 4)	
Range (ng/mL)	10–2000
Determination coefficient (R^2^)	0.9996 ± 0.0002
Slope a ± Δa	0.0003 ± 0.0000
Intercept b ± Δb	−0.0042 ± 0.0024
LLOQ (ng/mL)	10.0

**Table 2:** Validation parameters for the developed method. Stability of lamotrigine at four QC concentrations after storage at 8 °C and −21 °C, expressed as % loss.

QC (ng/mL)	Intra-Day (CV%)	Inter-Day (CV%)	Extraction Recovery (%)	Absolute Recovery (%)	Stability (Difference %)	
					8 °C	−21 °C
10	12.97	14.39	101.82	99.48	−3.46	−0.82
100	7.18	10.18	100.45	100.03	−3.70	−0.64
800	2.50	6.62	95.42	93.53	−5.96	−1.08
1500	5.20	6.92	93.63	90.95	−4.63	−0.98

**Table 3:** Patient’s data. Concentrations of lamotrigine found in saliva of seven male patients treated with 50 mg of the substance.

Patient	Age	Lamotrigine (ng/mL)
1	48	320.7
2	52	461.8
3	56	228.6
4	46	1058.0
5	39	539.2
6	53	445.1
7	60	628.1

## Data Availability

The data presented in this study are available on request from the corresponding author.

## Abbreviations

The following abbreviations are used in this manuscript:

LC	liquid chromatography
DAD	diode array detector
CV	coefficient of variation
BD	bipolar disorder
LLE	liquid–liquid extraction
SPE	solid-phase extraction
QC	quality control
EMA	European Medicines Agency
LLOQ	lower limit of quantification
